# MyD88 Signaling Inhibits Protective Immunity to the Gastrointestinal Helminth Parasite *Heligmosomoides polygyrus*

**DOI:** 10.4049/jimmunol.1401056

**Published:** 2014-08-11

**Authors:** Lisa A. Reynolds, Yvonne Harcus, Katherine A. Smith, Lauren M. Webb, James P. Hewitson, Ewan A. Ross, Sheila Brown, Satoshi Uematsu, Shizuo Akira, David Gray, Mohini Gray, Andrew S. MacDonald, Adam F. Cunningham, Rick M. Maizels

**Affiliations:** * Institute of Immunology and Infection Research, University of Edinburgh, Edinburgh, EH9 3JT, United Kingdom;; † Centre for Immunity, Infection and Evolution, University of Edinburgh, Edinburgh, EH9 3JT, United Kingdom;; ‡ Medical Research Council Centre for Immune Regulation, Institute of Microbiology and Infection, School of Immunity and Infection, University of Birmingham, Birmingham, B15 2TT, United Kingdom;; § Division of Innate Immune Regulation, International Research and Development Center for Mucosal Vaccines, Institute of Medical Science, The University of Tokyo, Shirokanedai, Minato-ku, Tokyo, 108-8639, Japan;; ¶ Department of Host Defense, Research Institute for Microbial Diseases, Osaka University, Suita, Osaka 565-0871, Japan; and; ‖ Laboratory of Host Defense, World Premier Institute Immunology Frontier Research Center, Osaka University, Suita, Osaka 565-0871, Japan

## Abstract

Helminth parasites remain one of the most common causes of infections worldwide, yet little is still known about the immune signaling pathways that control their expulsion. C57BL/6 mice are chronically susceptible to infection with the gastrointestinal helminth parasite *Heligmosomoides polygyrus*. In this article, we report that C57BL/6 mice lacking the adapter protein MyD88, which mediates signaling by TLRs and IL-1 family members, showed enhanced immunity to *H. polygyrus* infection. Alongside increased parasite expulsion, MyD88-deficient mice showed heightened IL-4 and IL-17A production from mesenteric lymph node CD4^+^ cells. In addition, MyD88^−/−^ mice developed substantial numbers of intestinal granulomas around the site of infection, which were not seen in MyD88-sufficient C57BL/6 mice, nor when signaling through the adapter protein TRIF (TIR domain–containing adapter–inducing IFN-β adapter protein) was also ablated. Mice deficient solely in TLR2, TLR4, TLR5, or TLR9 did not show enhanced parasite expulsion, suggesting that these TLRs signal redundantly to maintain *H. polygyrus* susceptibility in wild-type mice. To further investigate signaling pathways that are MyD88 dependent, we infected IL-1R1^−/−^ mice with *H. polygyrus.* This genotype displayed heightened granuloma numbers compared with wild-type mice, but without increased parasite expulsion. Thus, the IL-1R–MyD88 pathway is implicated in inhibiting granuloma formation; however, protective immunity in MyD88-deficient mice appears to be granuloma independent. Like IL-1R1^−/−^ and MyD88^−/−^ mice, animals lacking signaling through the type 1 IFN receptor (i.e., IFNAR1^−/−^) also developed intestinal granulomas. Hence, IL-1R1, MyD88, and type 1 IFN receptor signaling may provide pathways to impede granuloma formation in vivo, but additional MyD88-mediated signals are associated with inhibition of protective immunity in susceptible C57BL/6 mice.

## Introduction

Intestinal helminth parasites are highly prevalent worldwide (1), yet little is understood of the signaling pathways that lead to their immune exclusion. *Heligmosomoides polygyrus* is a natural intestinal nematode parasite of mice that can be maintained in the laboratory. Adult worms reside in the small intestine, alongside commensal organisms and dietary Ags, and release eggs into the feces for onward transmission. This tractable model system has been widely used to study factors affecting susceptibility to helminth infection (2–5).

Following oral ingestion of *H. polygyrus* larvae, the small intestinal epithelial cell barrier is disrupted first as the parasite’s infective larvae enter the small intestinal submucosa (by 24 h post infection), and later as the adult worms emerge to take up residence in the intestinal lumen (by 10 d post infection) (4). In most strains of mice, *H. polygyrus* establishes a long-term chronic infection, associated with the expansion of regulatory T cells (Tregs) (6–10), which inhibit host effector responses. However, some mouse strains express immunity to primary infection, as measured by diminished release of eggs in the feces, and faster expulsion of adult worms between 14 and 28 d of infection (10–12). Immunity can also be generated in most genotypes by prior immunization with parasite-secreted Ags (13, 14) or by abbreviating primary infection through drug treatment (15, 16).

Around the submucosal sites of larval invasion, type 2 granulomas consisting primarily of macrophages and neutrophils form (16–18). The number of intestinal granulomas generally correlates with the resistance phenotype of mouse strains, with the most resistant genotypes developing greater granuloma numbers following infection, which persist well after adult worms have emerged into the intestinal lumen, and have even been expelled (10, 16, 19). However, it is not known whether granuloma formation is necessary or sufficient for immunity to infection.

*H. polygyrus* shares its intestinal niche with many commensal microorganisms, and its penetration of the epithelial barrier may cause aberrant host exposure to microbial stimuli. In this respect, it is interesting to note previous reports that germ-free mice are more resistant to infection with *H. polygyrus* than are conventionally raised mice (20–22), leading us to hypothesize that direct or indirect signals from the intestinal microbiota modulate the outcome of infection.

The immune system recognizes highly conserved bacterial components through pattern recognition receptors (PRRs), which include the TLRs, nucleotide-binding oligomerization domain protein-like receptors, and C-type lectin receptors. Recognition of intestinal bacteria by these PRRs is required to maintain gut homeostasis in conditions in which epithelial cell barrier function is lost. For example, deficiencies in PRR signaling lead to heightened morbidity and mortality after dextran sodium sulfate treatment to experimentally induce colitis (23–25). Following epithelial cell damage, inflammatory and reparative cytokines, including TNF and IL-6, that can mediate repair are induced, and it is the recognition of intestinal microbes that provides the trigger for production of these cytokines (23).

It is likely that during periods of epithelial barrier disruption by *H. polygyrus,* ligation of PRRs by the intestinal microbiota occurs, but how this immune stimulation affects the antiparasite immune response has not yet been considered. A recent study has shown that when the integrity of the gut epithelium is disrupted during *Toxoplasma gondii* infection, a microflora-specific T cell response is mounted (26). Thus, it is reasonable to assume that the presence of the microflora will influence the immune environment surrounding infective *H. polygyrus* larvae.

In this article, we set out to investigate how immunity to *H. polygyrus* was affected when C57BL/6 mice, which typically are chronically susceptible to infection with this parasite, lacked the adapter protein MyD88, a major mediator of signaling through TLRs (27) and IL-1 family members (28). Strikingly, we show that MyD88^−/−^ mice were rendered more resistant to *H. polygyrus*. This resistance was associated with a heightened Th2 response, as well as a dramatic increase in the number of granulomas being produced following *H. polygyrus* infection, which rarely formed in C57BL/6 wild-type control mice. We demonstrate that granuloma formation is inhibited by a pathway involving IL-1R1, type 1 IFNR signaling, and MyD88, but distinct MyD88-dependent mechanisms maintain susceptibility to helminth infection in wild-type mice.

## Materials and Methods

### Mice

C57BL/6, TLR2^−/−^, TLR4^−/−^, TLR9^−/−^, MyD88^−/−^, TIR domain–containing adapter–inducing IFN-β adapter protein (TRIF)^−/−^, type 1 IFN receptor (IFNAR1)^−/−^, and MyD88^−/−^IFNAR1^−/−^ mice were bred in house at the University of Edinburgh. IL-1R1^−/−^ mice were bred at the University of Oxford, and TLR5^−/−^ mice at the University of Birmingham, U.K. All transgenic mice were on a C57BL/6 background. To equalize the microbiota compositions prior to beginning each experiment, bedding was mixed between C57BL/6 and transgenic male mouse cages, and female C57BL/6 and transgenic mice were cohoused for at least 1 wk prior to, and throughout the duration of, each experiment. Mice were housed in individually ventilated cages, and all experiments performed complied with U.K. Home Office guidelines.

### Parasites and granuloma measurements

Mice were infected by oral gavage of 200 *H. polygyrus bakeri* third stage larvae, which were obtained from fecal cultures of *H. polygyrus*–infected mice. *H. polygyrus* egg counts were performed on fecal pellets using a McMaster 2 cell counter (Hawksley) at 2 wk and 4 wk following infection, and 4 wk following infection, intestinal adult worms and granulomas were enumerated. Granulomas were macroscopically counted along the entire length of the small intestinal tract and were not present in the large intestine.

### Cell staining and flow cytometry

Mesenteric lymph nodes (MLNs) were manually dissected, and single-cell suspensions were made by passing cells through a 70μM cell strainer in RPMI 1640 media (Life Technologies) containing 10% FCS (Hyclone), 2 mM l-glutamine (Life Technologies), and 100 U/ml penicillin and 100 μg/ml streptomycin (Life Technologies).

Cells were stained directly ex vivo for Foxp3 measurements or, for intracellular cytokine measurements, restimulated with 0.5 μg/ml PMA and 1 μg/ml ionomycin for 3.5 h, with 10 μg/ml brefeldin A included for the final 2.5 h. Cells were stained for 20 min at 4°C with Abs to the surface marker CD4 (RM4-5; BD Pharmingen), and CD103 conjugated to biotin (M290; BD Pharmingen) followed by PerCP-streptavidin (BD Pharmingen). Cells were fixed according to the manufacturer’s instructions with Fix/Perm (eBiosciences) for Foxp3 measurements, or Cytofix/Cytoperm (BD) for intracellular cytokine measurements, and then stained for 20 min at 4°C with Abs to Foxp3 (FJK-16s; eBioscience), IL-4 (11B11; BioLegend), IFN-γ (XMG1.2; BioLegend), IL-17A (TC11-18H10.1; BioLegend), or the relevant isotype controls. Marker expression was measured on FACSCanto (BD) or LSR II (BD) flow cytometers, and data were analyzed using FlowJo software (TreeStar).

### Statistics

Data were first examined for normality. Where data were normally distributed, and comparisons between two groups were being made, unpaired *t* tests were performed, and where comparisons between multiple groups were being made, one-way ANOVA tests followed by Tukey tests were used. In situations when data were not normally distributed, Mann–Whitney tests were used to compare two groups or, for comparisons between multiple groups, Kruskal–Wallis tests followed by Dunn tests were used.

Average bars shown on graphs indicate the mean value for parametric data, or the median value for nonparametric data. The *p* values are as follows: *p ≤ 0.05, **p ≤ 0.01, ***p ≤ 0.001.

Data from multiple experiments were pooled where indicated, and only when no statistical differences existed between separate data sets.

## Results

### MyD88 deficiency renders mice more resistant to *H. polygyrus* than wild-type C57BL/6 mice

**FIGURE 1.**
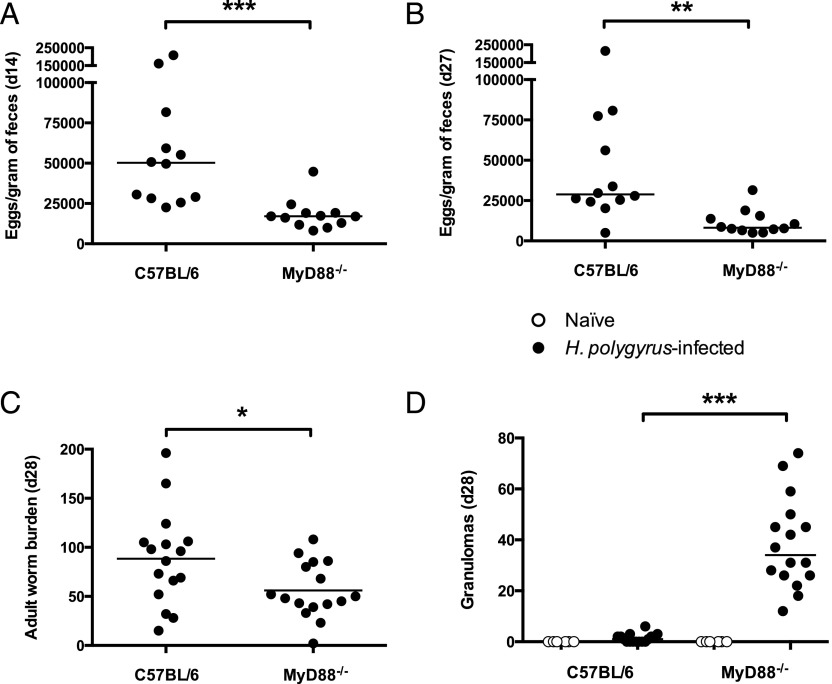
MyD88 deficiency renders mice more resistant to *H. polygyrus* than wild-type C57BL/6 mice. C57BL/6 and MyD88^−/−^ mice were left naive or were infected with 200 *H. polygyrus* third stage larvae. (**A)** *H. polygyrus* eggs per gram of feces taken 14 d post infection. (**B**) *H. polygyrus* eggs per gram of feces taken 27 d post infection. (**C**) Adult *H. polygyrus* numbers recovered from the intestinal tract 28 d post infection. (**D)** Number of granulomas formed along the small intestinal tract 28 d post infection. Data shown in (A) and (B) are pooled from two independent experiments, each with four to eight mice per group; data shown in (C) and (D) are pooled from three independent experiments, each with four to eight mice per group. *p ≤ 0.05, **p ≤ 0.01, ***p ≤ 0.001.

To investigate whether PRR signaling modulates the outcome of a helminth infection, we first compared mice lacking the adapter protein MyD88, through which many TLRs signal (27), with wild-type C57BL/6 mice for their susceptibility to *H. polygyrus*. Although wild-type C57BL/6 mice are chronically susceptible to this parasite, a lack of MyD88 signaling was found to render mice markedly more resistant to infection, with a lower *H. polygyrus* egg output seen at days 14 and 27 post infection (Fig. 1A, 1B), an indication of lower worm fitness, which correlates with stronger immune responsiveness (10). In addition, significantly fewer adult worms remained in the MyD88-deficient host 28 d following infection (Fig. 1C).

A striking phenotype in MyD88^−/−^ mice was the increased number of granulomas that formed along the small intestinal tract following *H. polygyrus* infection, which were rarely observed at this time point in wild-type C57BL/6 mice (Fig. 1D). Granuloma formation has previously been associated with more rapid helminth expulsion, with increased numbers of granulomas evident in more genetically resistant mouse strains (10, 19).

To determine whether MyD88 signaling by hematopoietic or nonhematopoietic cells is important for controlling immunity to *H. polygyrus*, bone marrow chimeras were generated in which either the hematopoietic or the nonhematopoietic compartment lacked MyD88 expression. Neither MyD88 deficiency solely within hematopoietic, nor within nonhematopoietic cells, was sufficient to result in the heightened immunity of mice completely deficient in MyD88, suggesting that MyD88 signaling by both compartments contributes to the susceptibility of C57BL/6 mice to *H. polygyrus* (Supplemental Fig. 1).

### Mice singly deficient in specific TLRs harbor *H. polygyrus* burdens similar to those in wild-type C57BL/6 mice

MyD88 is used for signaling by TLRs (27). To address whether the increased resistance to *H. polygyrus* in MyD88^−/−^ mice was due to lack of signaling through a single, specific TLR, mice deficient in individual TLRs known to sense bacterial products were assessed for their susceptibility to *H. polygyrus*.

**FIGURE 2.**
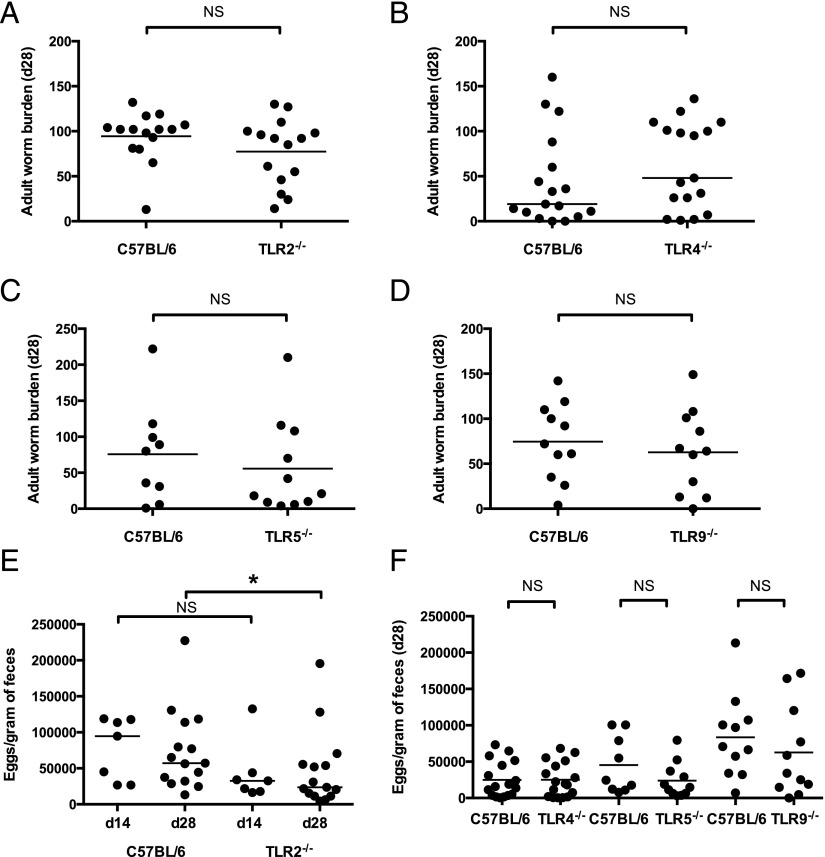
Mice singly deficient in specific TLRs harbor *H. polygyrus* burdens similar to those in wild-type C57BL/6 mice. Mice of each genotype were infected with 200 *H. polygyrus* third stage larvae. (**A–D**) Adult *H. polygyrus* numbers recovered from the intestinal tract 28 d post infection are shown in C57BL/6 and (A) TLR2^−/−^ mice, data shown pooled from four independent experiments, each with three to four mice per group; (B) TLR4^−/−^ mice, data shown pooled from three independent experiments, each with five to six mice per group; (C) TLR5^−/−^ mice, data shown pooled from two independent experiments, each with four to six mice per group; and (D) TLR9^−/−^ mice, data shown pooled from two independent experiments, each with five to six mice per group and representative of results from five independent experiments. (**E**) *H. polygyrus* eggs per gram of feces taken 14 and 28 d post infection in C57BL/6 and TLR2^−/−^ mice; data shown at day 14 were pooled from two independent experiments, each with three to four mice per group, and data shown at day 28 were pooled from four independent experiments, each with two to four mice per group. Statistics shown indicate comparisons made between genotypes at the same time point. (**F)** *H. polygyrus* eggs per gram of feces taken 28 d post infection in C57BL/6, TLR4^−/−^, TLR5^−/−^, and TLR9^−/−^ mice; data shown pooled from two to three independent experiments, each with four to six mice per group. *p ≤ 0.05.

The ability of MyD88-deficient mice to expel *H. polygyrus* more rapidly was not reproduced in the absence of TLR2 (Fig. 2A), TLR4 (Fig. 2B), TLR5 (Fig. 2C), or TLR9 (Fig. 2D) alone, leading to the possibility that TLRs signal redundantly to maintain *H. polygyrus* susceptibility on the C57BL/6 background. However, in TLR2^−/−^ mice *H. polygyrus* showed reduced egg output by day 28 post infection, compared with wild-type C57BL/6 controls (Fig. 2E), although considerable variability in egg output was seen in these mice. In contrast, neither TLR4^−/−^, TLR5^−/−^, nor TLR9^−/−^ mice showed significant differences in *H. polygyrus* egg output, compared with wild-type controls (Fig. 2F).

### MyD88^−/−^ mice mount a stronger CD4^+^ T cell IL-4 and IL-17A response following *H. polygyrus* infection

**FIGURE 3.**
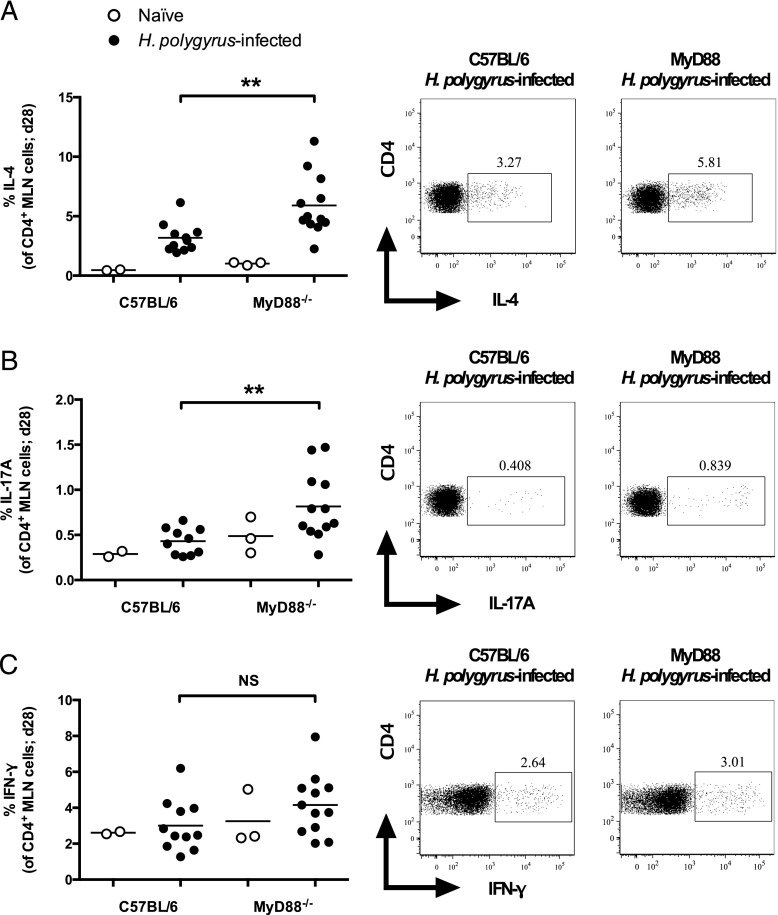
MyD88^−/−^ mice mount stronger CD4^+^ T cell IL-4 and IL-17A responses following *H. polygyrus* infection. C57BL/6 and MyD88^−/−^ mice were left naive or infected with 200 *H. polygyrus* third stage larvae. At 28 d following infection, MLN cells were isolated and restimulated with PMA/ionomycin and brefeldin A, after which cells were stained as indicated and cytokine production was measured by flow cytometry. (**A**) Percentage of IL-4–producing cells among CD4^+^, live lymphocyte cells, and representative dot plots. (**B**) Percentage of IL-17A–producing cells among CD4^+^, live lymphocyte cells, and representative dot plots. (**C**) Percentage of IFN-γ–producing cells among CD4^+^, live lymphocyte cells, and representative dot plots. Data shown in (A)**–**(C) are pooled from two independent experiments, each with four to seven *H. polygyrus*-infected mice per group; naive mice were examined in only one of these experiments. Statistics shown indicate comparisons between infected groups. **p ≤ 0.01.

We next investigated how T cell responses, which play an important role in mediating immunity to a primary *H. polygyrus* infection (4, 29), differed between wild-type C57BL/6 and MyD88-deficient mice. At 28 d post *H. polygyrus* infection, levels of IL-4 and of IL-17A being produced by CD4^+^ T cells in the MLN cells of MyD88-deficient mice were higher than in wild-type C57BL/6 counterparts (Fig. 3A, 3B), whereas IFN-γ levels were similar between infected groups (Fig. 3C). IL-4 is recognized as one of the most critical cytokines for mediating protective immunity to *H. polygyrus* (4, 30); however, Ab-mediated depletion of IL-17A has recently been found to have little effect on the outcome of primary infection with this parasite (31). Despite the reduced *H. polygyrus* egg output in TLR2^−/−^ mice compared with wild-type mice (Fig. 2E), MLN CD4^+^ T cells from naive and 28-d *H. polygyrus*–infected TLR2^−/−^ mice produced levels of IL-4, IL-17A, and IFN-γ comparable to those from wild-type mice (Supplemental Fig. 2**)**.

### TLR signaling contributes differentially to Treg proportions following *H. polygyrus* infection

**FIGURE 4.**
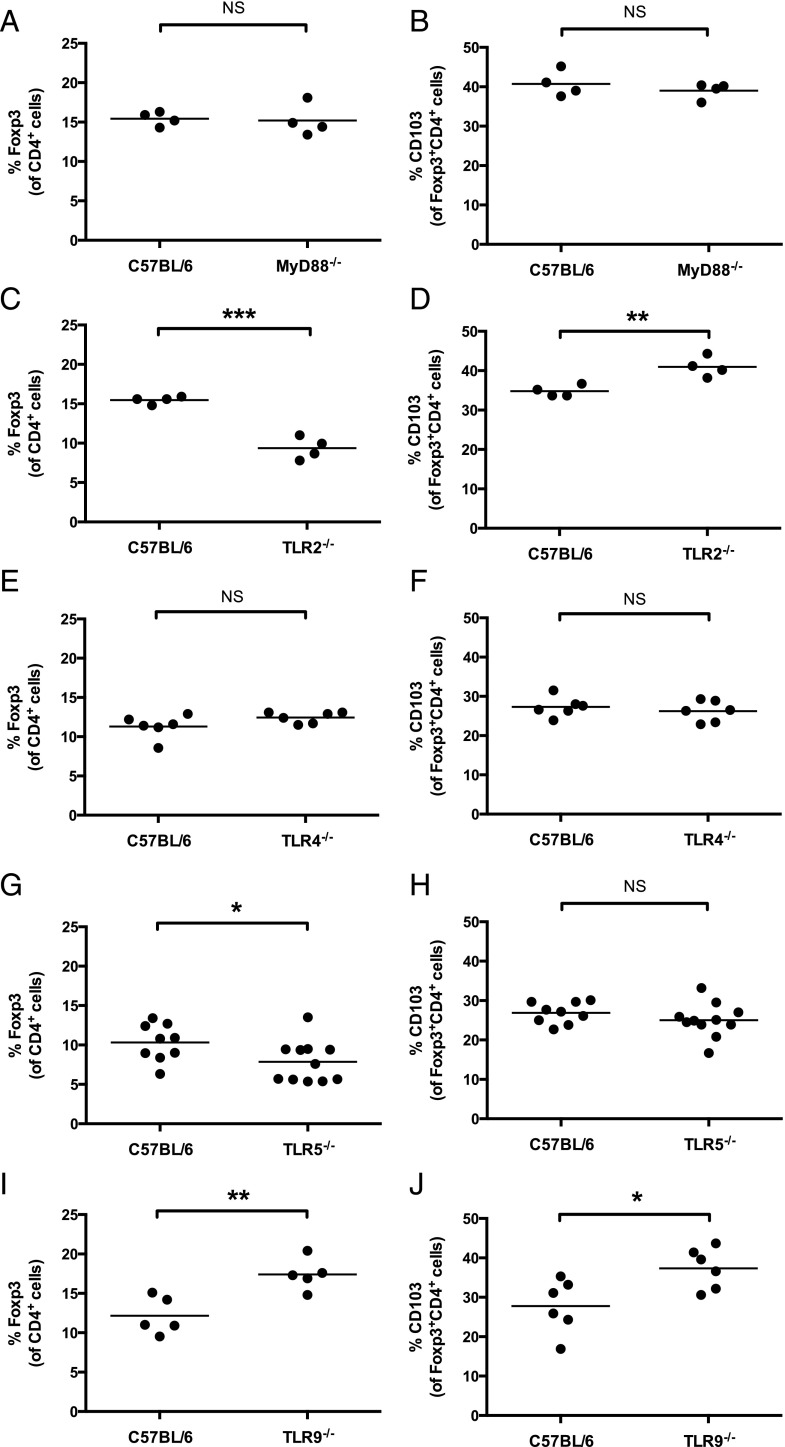
TLR signaling contributes differentially to MLN Treg proportions following *H. polygyrus* infection. All mice were infected with 200 *H. polygyrus* third stage larvae. At 28 d following infection, MLN cells were isolated, stained as indicated, and analyzed by flow cytometry. (**A**, **C**, **E**, **G**, **I**) Percent of Foxp3^+^ cells among CD4^+^ lymphocytes in wild-type C57BL/6 and (A) MyD88^−/−^, (C) TLR2^−/−^, (E) TLR4^−/−^, (G) TLR5^−/−^, and (I**)** TLR9^−/−^ mice. (**B**, **D**, **F**, **H**, **J**) Percent of CD103^+^ cells among Foxp3^+^CD4^+^ lymphocytes in wild-type C57BL/6 and (B) MyD88^−/−^, (D) TLR2^−/−^, (F) TLR4^−/−^, (H) TLR5^−/−^, and (J) TLR9^−/−^ mice. Data shown in (A) and (B) are representative of results from three independent experiments, each with four to seven mice per group; data in (C) and (D) are representative of results from two independent experiments, each with four mice per group; data in (E) and **(**F) from one experiment with six mice per group; data in (G) and (H) are pooled from two independent experiments, each with four to six mice per group; and data in (I) and (J) are representative of results from five independent experiments, each with five to seven mice per group. *p ≤ 0.05, **p ≤ 0.01, ***p ≤ 0.001.

As MyD88-deficient mice produced more signature cytokines of both Th2 and Th17 subsets following *H. polygyrus* infection, we then examined whether the proportions and activation status of Tregs were abnormal in these mice. Surprisingly, no deficiencies in Foxp3^+^CD4^+^ T cell proportions, or in their expression of CD103, were seen in the MLNs of MyD88-deficient mice (Fig. 4A, 4B**)**.

Dysregulation of Treg proportions were, however, seen in singly TLR-deficient mice. Both TLR2- and TLR5-deficient mice had a lower proportion of Foxp3^+^CD4^+^ MLN T cells following *H. polygyrus* infection than did wild-type C57BL/6 mice (Fig. 4C, 4G). A greater proportion of Foxp3^+^CD4^+^ MLN T cells in TLR2-deficient mice expressed CD103 (Fig. 4D) than in wild-type C57BL/6, whereas CD103 expression levels on Foxp3^+^CD4^+^ MLN T cells were similar between wild-type C57BL/6 and TLR5-deficient mice (Fig. 4H). Conversely, TLR9-deficient mice had a higher proportion of Foxp3^+^CD4^+^ MLN T cells following *H. polygyrus* infection than did wild-type C57BL/6 mice (Fig. 4I), and expression of CD103 among these cells was also higher than in wild-type mice (Fig. 4J). TLR4-deficient mice displayed no differences from wild-type C57BL/6 mice in Foxp3^+^CD4^+^ MLN T cell proportions or CD103 expression following *H. polygyrus* infection (Fig. 4E, 4F). MLN total cell counts following *H. polygyrus* infection were comparable to wild-type C57BL/6 mice cell numbers for all genotypes examined (data not shown).

Thus, signaling through individual TLRs contributes differentially to Treg proportions and CD103 expression levels following infection, resulting in no overall differences in Treg phenotype from wild-type C57BL/6 mice when TLR signaling is ablated in MyD88-deficient mice.

### TRIF modulates granuloma formation but not antiparasite immunity

**FIGURE 5.**
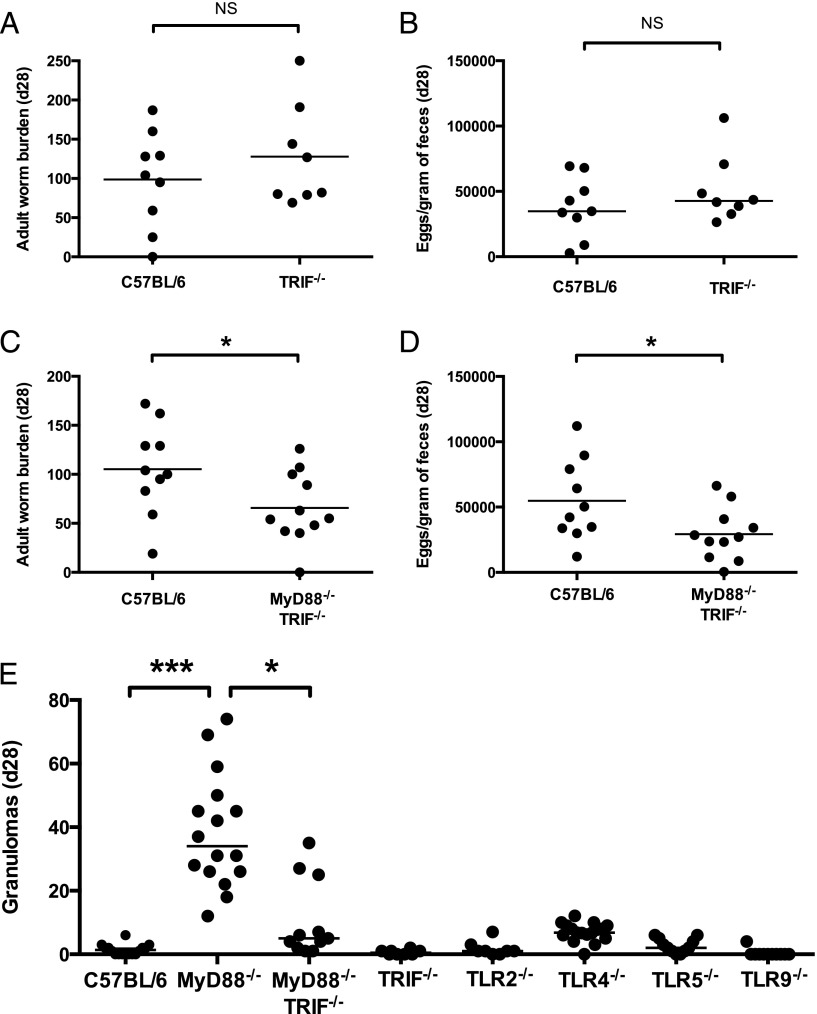
MyD88^−/−^, but not MyD88^−/−^TRIF^−/−^, mice display heightened granuloma formation in response to *H. polygyrus* infection. Mice of each genotype were infected with 200 *H. polygyrus* third stage larvae. (**A**) Adult *H. polygyrus* numbers recovered from the intestinal tract of C57BL/6 and TRIF^−/−^ mice 28 d post infection. (**B**) *H. polygyrus* eggs per gram of feces from C57BL/6 and TRIF^−/−^ mice taken 28 d post infection. (A and B) Data shown pooled from two independent experiments, each with three to five mice per group. (**C**) Adult *H. polygyrus* numbers recovered from the intestinal tract of C57BL/6 and MyD88^−/−^TRIF^−/−^ mice 28 d post infection. (**D**) *H. polygyrus* eggs per gram of feces from C57BL/6 and MyD88^−/−^TRIF^−/−^ mice taken at 28 d post infection. (C and D) Data shown pooled from three independent experiments, each with two to six mice per group. (**E**) Number of granulomas formed along the small intestinal tract in the indicated mouse genotypes 28 d following *H. polygyrus* infection. Data shown for each genotype are pooled from two or three independent experiments, and experiments for each genotype were performed separately. A statistical comparison is shown only between the C57BL/6, MyD88^−/−^, and MyD88^−/−^TRIF^−/−^ groups. *p ≤ 0.05, ***p ≤ 0.001.

MyD88^−/−^ mice retain the ability to respond to certain bacterial signals, as TLR4, which recognizes LPS (32), can signal independently of MyD88 through the TRIF adapter protein (33). Thus, only mice deficient in both MyD88 and TRIF are unable to respond to bacterial ligands through TLRs; hence we tested TRIF-deficient and MyD88 × TRIF doubly deficient mice for their susceptibility to *H. polygyrus* infection. Unlike MyD88^−/−^ mice, TRIF^−/−^ mice did not show increased parasite expulsion, with adult worm numbers (Fig. 5A) and egg burdens (Fig. 5B) matching those of the C57BL/6 wild-type. In contrast, MyD88^−/−^TRIF^−/−^ mice were similar to their MyD88^−/−^ counterparts, with reduced *H. polygyrus* adult worm survival (Fig. 5C) and egg production (Fig. 5D), compared with wild-type mice, by day 28 post infection, confirming that MyD88 is a key adapter protein in host susceptibility to *H. polygyrus*.

In these experiments, individual TLR-deficient and TRIF-deficient mice were also examined for the presence of granulomas following *H. polygyrus* infection. No mice deficient in individual TLR or TRIF molecules produced the high number of granulomas seen in MyD88^−/−^ mice (Fig. 5E). Surprisingly, MyD88^−/−^TRIF^−/−^ mice developed far fewer granulomas than mice lacking only MyD88 (Fig. 5E), suggesting that TRIF is required for the high numbers of granulomas observed in the absence of MyD88 or that it interacts with the pathways affected by loss of MyD88.

### Heightened granuloma numbers in MyD88-deficient mice, but not heightened expulsion, can be attributed to a lack of IL-1R signaling

**FIGURE 6.**
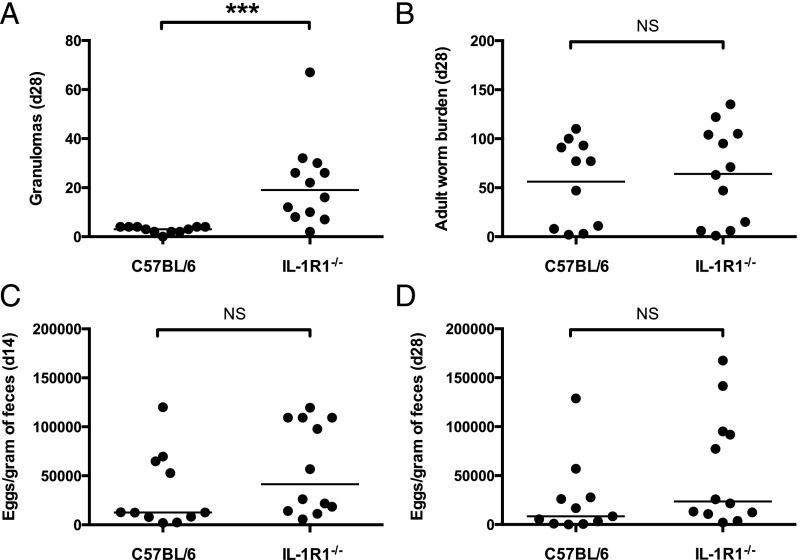
Heightened granuloma numbers, but not heightened expulsion, can be attributed to a lack of IL-1R1 signaling. C57BL/6 and IL-1R1^−/−^ mice were infected with 200 *H. polygyrus* third stage larvae. Data shown are pooled from two independent experiments, each with five to six mice per group. (**A**) Number of granulomas formed along the small intestinal tract 28 d following *H. polygyrus* infection. (**B**) Adult *H. polygyrus* numbers recovered from the intestinal tract 28 d post infection. (**C**) *H. polygyrus* eggs per gram of feces taken 14 d post infection. (**D**) *H. polygyrus* eggs per gram of feces taken 28 d post infection. ***p ≤ 0.001.

In addition to mediating signaling from TLRs, MyD88 is also a critical adapter protein for signaling by the IL-1 family members IL-1α, IL-1β, IL-18, and IL-33 (28). Because individual TLR-deficient mice did not recapitulate the increased resistance of MyD88-deficient mice to *H. polygyrus*, and IL-1 has recently been reported to promote susceptibility to this parasite (34), we next examined whether mice lacking IL-1R signaling (IL-1R1^−/−^) showed heightened immunity to this parasite. Of interest, IL-1R1^−/−^ mice, consistent with previously reported findings in IL-1β^−/−^ mice (34), produced high numbers of granulomas in response to infection (Fig. 6A). However, adult *H. polygyrus* burdens 28 d post infection, and egg output at days 14 and 28 post infection, were similar between IL-1R1^−/−^ and wild-type C57BL/6 mice (Fig. 6B–D), suggesting that heightened granuloma formation does not contribute to increased parasite expulsion and that the phenotype of IL-1R1 deficiency is not as profound as that reported for mice lacking IL-1β (34).

### Type 1 IFN signaling may act as downstream inhibitor of granuloma formation

**FIGURE 7.**
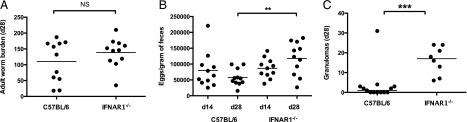
Type 1 IFN signaling represses granuloma formation. All mice were infected with 200 *H. polygyrus* third stage larvae. (**A**) Adult *H. polygyrus* numbers recovered from the intestinal tract of C57BL/6 and IFNAR1^−/−^ mice 28 d post infection; data shown are representative of two experiments, each with five to six mice per group. (**B**) *H. polygyrus* eggs per gram of feces taken 14 and 28 d post infection in C57BL/6 and IFNAR1^−/−^ mice; data shown are representative of two experiments, each with five to six mice per group. (**C**) Number of granulomas formed along the small intestinal tract 28 d following *H. polygyrus* infection. Data shown are pooled from two experiments, each with two to six mice per group. **p ≤ 0.01, ***p ≤ 0.001.

Type 1 IFNs (IFN-α/IFN-β) can be produced in response to stimulation of both MyD88- and TRIF-dependent pathways (35), and signal through a heterodimeric receptor consisting of IFN-αR1 (i.e., IFNAR1) and IFN-αR2c (36). To test whether granuloma formation was affected by a loss of type 1 IFN signaling, we infected IFNAR1^−/−^ mice (37) with *H. polygyrus*. IFNAR1^−/−^ mice did not share the heightened parasite immunity phenotype shown by MyD88^−/−^ mice, as similar numbers of adult worms were recovered 28 d post infection as in wild-type C57BL/6 mice (Fig. 7A), and *H. polygyrus* egg production was even greater in IFNAR1^−/−^ mice than in wild-type controls 28 d following infection (Fig. 7B). However, this genotype showed increased formation of granulomas (Fig. 7C), indicating that like MyD88, type 1 IFNs inhibit this process in wild-type mice.

## Discussion

In this study, we investigated the outcome of a primary *H. polygyrus* infection in mice deficient in MyD88 signaling, and found that they were more resistant to infection than wild-type C57BL/6 mice and had a higher frequency of IL-4–producing CD4^+^ T cells following infection. In addition, MyD88-deficient mice developed large numbers of granulomas along the intestinal wall in response to infection. Intestinal granulomas that form in response to *H. polygyrus* infection are enriched in alternatively activated macrophages and neutrophils (10, 16), and are more frequent in more resistant strains of mice (10, 19).

Many helminth parasites are able to establish chronic infections in mammals by manipulation of host immunity, which is largely attributed to the secretion and action of parasite excretory–secretory proteins (38). One pathway by which helminths can modulate host signaling is through interference with mammalian TLR signaling (39–41). Several examples of helminth products that can bind to TLRs and mediate signaling through these receptors have been documented, such as dsRNA from *Schistosoma mansoni* eggs, which binds TLR3, leading to NF-κB activation (42), and the lipid lysophosphatidylserine extracted from *S. mansoni* eggs or adult worms, which stimulates TNF-α and IL-10 production via TLR2 (43). Similarly, the excretory–secretory product ES-62 from the rodent filarial nematode *Acanthocheilonema viteae* signals via TLR4 to cause IL-12 and TNF-α production (44), and the *S. mansoni* Lewis^X^-containing egg carbohydrate lacto-*N*-fucopentaose III binds TLR4 to drive MAPK activation and IL-4 production (45). LPS signaling through TLR4 does not typically induce IL-4 production, and how helminth products induce differential cytokine production following TLR ligation/activation is yet to be resolved. A potential mechanism by which helminth products can skew downstream responses of TLR signaling is through the additional engagement of coreceptors (46).

Infection with *H. polygyrus* may result in TLR signaling not only through secretory molecules but also by increasing the exposure of immune cells to bacterial ligands. In particular, when *H. polygyrus* larvae disrupt the epithelial cell barrier during migration through to the submucosa, they likely facilitate bacterial translocation and contact with host cells through to the serosal layer of the gut. If so, signals from bacterial ligands may significantly impair the ability of the host to mount a protective response to helminth infection.

The increased *H. polygyrus* expulsion seen in MyD88-deficient mice was not replicated in any individual TLR-deficient mouse examined, although *H. polygyrus* egg output was reduced by day 28 post infection in TLR2-deficient mice, suggesting that signaling through this receptor partially regulates immunity to *H. polygyrus*. Deficiencies in individual TLRs did, however, alter Treg proportions following infection. Consistent with the results described in this article, TLR2 and TLR5 engagement has been previously shown to expand Tregs (47–49), whereas TLR9 stimulation has been shown to limit Treg function (50). TLR4 stimulation enhances Treg survival and proliferation in vitro (51), but in these in vivo experiments, TLR4 deficiency did not affect Treg proportions. Infection status likely determines the importance of TLR signaling in controlling Treg cell frequencies; TLR2-deficient mice have equal proportions of Foxp3^+^CD4^+^ cells at the MLN site at steady state (52), yet during the *H. polygyrus* infections performed in this study, and during *Candida albicans* infection (53), proportions are reduced, compared with wild-type mice. These findings highlight that TLR control of Treg expansion and proliferation is dependent on both site and context (54).

Although no individual TLR gene deficiency recapitulated the phenotype of the MyD88^−/−^ mouse, we found that mice deficient in IL-1R1, which signals through MyD88, did reproduce the granulomatous response following *H. polygyrus* infection, suggesting that signaling through the IL-1R1 represses granuloma formation in MyD88-sufficient mice. Consistent with this finding, IL-1β–deficient mice have been reported to develop more, and longer-lasting, granulomas during *H. polygyrus* infection (34). However, despite their enhanced granulomas, IL-1R1^−/−^ mice displayed no differences from wild-type mice in adult worm expulsion, indicating that granuloma formation is not a key element of immunity to this parasite. The possibility cannot be excluded, however, that the cellular components of *H. polygyrus*–induced granulomas differ in quality between MyD88-deficient and IL-1R1–deficient mice.

If granulomas are not a mechanism of immunity to *H. polygyrus*, they may instead form to repair damage caused by either the migrating parasite or the resultant immune response (55). Together, these findings lead to the intriguing hypothesis that a component of the microbiota, signaling via IL-1R and MyD88, may function to inhibit granuloma formation in response to helminth infection. The composition of the intestinal microbiota may thus be a factor in controlling a granulomatous response to *H. polygyrus* infection.

These data also raise broader questions about the role of IL-1 signaling during helminth infections, as IL-1α and IL-1β appear to promote protective Th2 responses against the helminth parasite *Trichuris muris* (56), yet the IL-1R is reportedly not necessary for protective immunity (57). Moreover, a recent study reported that IL-1β–deficient mice are more resistant to *H. polygyrus* (34), again indicating that a ligand-dependent, but IL-1R–independent, pathway yet to be defined is important in immunity to helminths.

The IL-1 family members IL-18 and IL-33 also signal in a manner that is dependent on MyD88 (28, 58). IL-33 has previously been shown to promote type 2 cytokine production and helminth parasite expulsion (59–61); therefore, it is unlikely that a lack of IL-33 is responsible for the heightened immunity of MyD88^−/−^ mice. In fact, IL-33R (ST2)–deficient mice show *H. polygyrus* worm burdens similar to those in wild-type BALB/c controls (34), confirming that this alarmin cytokine is not essential for immunity to *H. polygyrus*, although important in maximizing rejection of other intestinal helminths.

It will be important, however, for future work to examine whether a lack of IL-18 recapitulates the heightened resistance of MyD88^−/−^ mice, as during infection with *T. muris*, IL-18 has been shown to promote susceptibility to infection through the inhibition of Th2 cytokines (62). In some contexts, however, IL-18 has been shown to promote Th2 cytokine production (59), highlighting that the actions of this cytokine are highly dependent on the surrounding cytokine environment and on the genetic background of the host (63, 64). Signaling through inflammasome complexes is required for the release of mature IL-1 and IL-18 (65); thus, modulation of inflammasome signaling to control IL-1 or IL-18 release may be a mechanism by which the microbiota could influence the immune response to helminth infections.

It has previously been reported that DCs unable to signal using MyD88 are impaired in the production of type 1 IFNs (66). We show that mice deficient in type 1 IFN signaling (IFNAR1^−/−^ mice) produce the high levels of intestinal granulomas also seen in MyD88^−/−^ mice, raising the possibility that following MyD88 stimulation, type 1 IFNs signal via IFNAR1 to inhibit granuloma formation in wild-type mice. Intriguingly, we also show that ablation of signaling through the adapter protein TRIF, on a MyD88^−/−^ background, abolished the heightened granuloma formation, suggesting that stimulation of TRIF promotes the formation of intestinal granulomas, through an as-yet-uncharacterized pathway.

With all this information taken together, this report demonstrates the importance of both TLR-dependent and -independent signals through MyD88 in the control of host resistance following helminth infection. A previous report demonstrated that mice deficient in MyD88 show heightened expulsion of *T. muris* (67), suggesting that inhibition of immunity by MyD88-mediated signals may be a common mechanism to allow intestinal colonization by helminth parasites. An absence of MyD88 signaling in our study resulted in heightened *H. polygyrus* expulsion, a phenotype that was partially replicated in TLR2-deficient mice, but not in mice deficient in TLR4, TLR5, or TLR9. This observation raises two possibilities: that signals through individual TLRs redundantly maintain helminth susceptibility or that a TLR-independent pathway is key to the heightened immunity of MyD88-deficient mice. Owing to recent evidence that the presence of the intestinal microbiota is key to maintaining the lifecycle and establishment of helminth parasites within mammalian hosts (68), and with new data showing *H. polygyrus* infection is enhanced by specific commensal bacteria (69), it will be critical to further understand the signaling pathways by which interactions between the microbiota, the immune system, and parasites are controlled.

## Supplementary Material

Data Supplement
