# Supplementary material for: MyD88 Signaling Inhibits Protective Immunity to the Gastrointestinal Helminth Parasite Heligmosomoides polygyrus
Source: J Immunol. 2014 Aug 11;193(6):2984–93. doi: 10.4049/jimmunol.1401056 (PMC4157852; doi:10.4049/jimmunol.1401056)
Supplement: Data Supplement [file supp_193_6_2984__index.html]

MyD88 Signaling Inhibits Protective Immunity to the Gastrointestinal Helminth Parasite Heligmosomoides polygyrus — MyD88 Signaling Inhibits Protective Immunity to the Gastrointestinal Helminth Parasite Heligmosomoides polygyrus — Data Supplement 

# MyD88 Signaling Inhibits Protective Immunity to the Gastrointestinal Helminth Parasite *Heligmosomoides polygyrus*

## Data Supplement

**Files in this Data Supplement:**

- Supplemental Figures 1 (PDF)
