# Supplementary material for: MyD88 Signaling Inhibits Protective Immunity to the Gastrointestinal Helminth Parasite Heligmosomoides polygyrus
Source: J Immunol. 2014 Aug 11;193(6):2984–93. doi: 10.4049/jimmunol.1401056 (PMC4157852; doi:10.4049/jimmunol.1401056)
Supplement: Data Supplement [file 1401056_JI_1401056_Supplemental_Figures_1.pdf]

Supplemental Figure 1

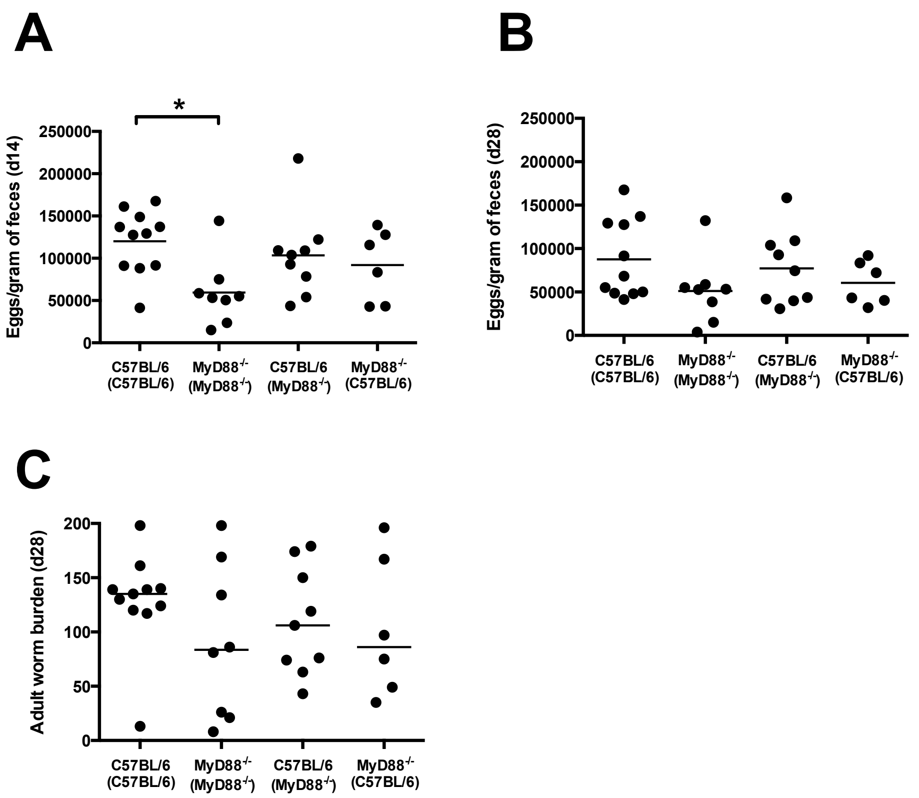

SUPPLEMENTAL FIGURE 1. MyD88-deficiency is required both in hematopoietic and non-hematopoietic compartments for increased immunity to *H. polygyrus*. Five days prior to irradiation, all recipient mice were placed on water containing 0.1 mg/ml Enrofloxacin (Baytril;Bayer), which they were kept on for four weeks following irradiation. Recipient mice were given a lethal radiation dose of 980 radiation absorbed dose. 24 hours following irradiation, mice were reconstituted with 2-5 million bone marrow cells from donor mice by injection into the tail vein. To prepare cells for reconstitution, donor mice were sacrificed, and bone marrow cells extracted into PBS. Red blood cells were lysed by incubating cell suspensions in red blood cell lysis buffer (Sigma-Aldrich) at room temperature for 5 minutes. Cells were washed twice in PBS, and depleted of $CD90^+$ cells using CD90.2 microbeads (Miltenyi Biotech) and MACS negative selection columns (Miltenyi Biotech) using the manufacturer's recommended instructions. The genotypes of bone marrow cells used for reconstitution are shown in the text and figures in parentheses. Eight weeks following reconstitution, all mice were infected with 200 *H. polygyrus* L3s. Data shown are pooled from 2 independent experiments, each with 2-6 mice per group. (**A**) *H. polygyrus* eggs per gram of feces taken 14 days post-infection. (**B**) *H. polygyrus* eggs per gram of feces taken 28 days post-infection. (**C**) Adult *H. polygyrus* numbers recovered from the intestinal tract 28 days post-infection.

Supplemental Figure 2

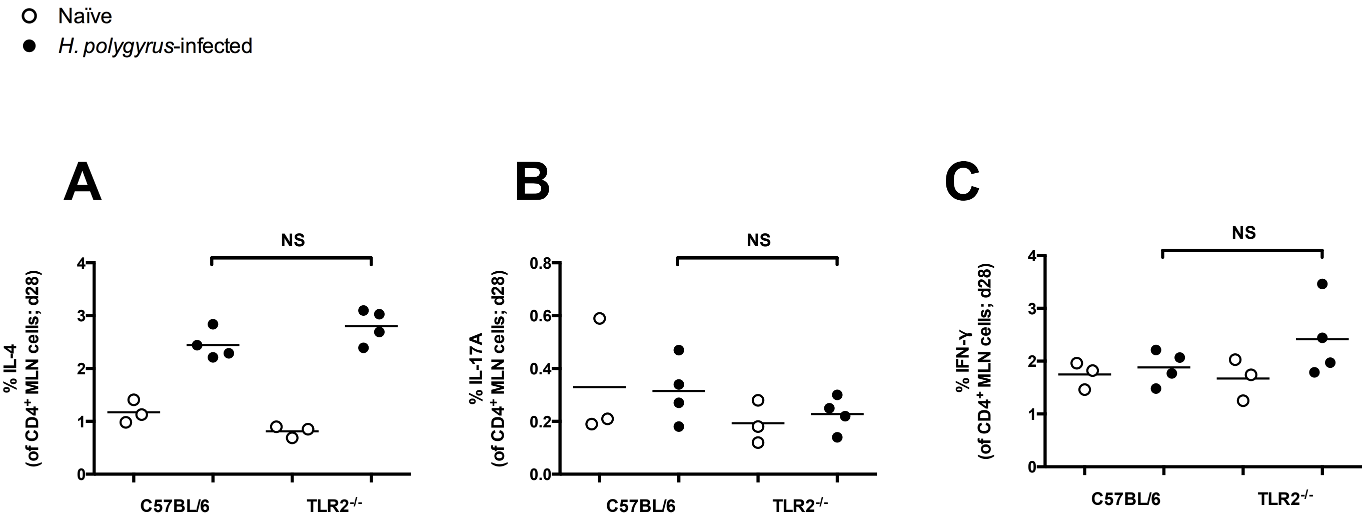

SUPPLEMENTAL FIGURE 2. TLR2$^{-/-}$ mice mount a comparable CD4^{+} T cell IL-4 response to wildtype mice following *H. polygyrus* infection. C57BL/6 and TLR2$^{-/-}$ mice were left naïve or infected with 200 *H. polygyrus* third stage larvae. 28 days following infection, MLN cells were isolated and restimulated with PMA/Ionomycin and Brefeldin A, after which cells were stained as indicated and cytokine production was measured by flow cytometry. Data shown are from 1 experiment with 3-4 mice per group, and are representative of the results from 2 independent experiments. Statistics shown indicate comparisons between infected groups. (**A**) Percentage of IL-4-producing cells amongst CD4^{+}, live lymphocyte cells. (**B**) Percentage of IL-17A-producing cells amongst CD4^{+}, live lymphocyte cells. (**C**) Percentage of IFN-γ-producing cells amongst CD4^{+}, live lymphocyte cells.
